# Laparoscopic transabdominal approach for resection of presacral epidermoid cyst in an obese man: a case report

**DOI:** 10.1186/s40792-024-01924-8

**Published:** 2024-05-13

**Authors:** Naohiro Yoshida, Koki Nakamura, Takahiro Shigaki, Kenji Fujiyoshi, Kenichi Koushi, Takefumi Yoshida, Taro Isobe, Naoki Mori, Tomoya Sudo, Hisamune Sakai, Toru Hisaka, Nobuya Ishibashi, Jun Akiba, Fumihiko Fujita

**Affiliations:** 1 Department of Surgery, Kurume University School of Medicine, 67, Asahi-machi, Kurume-shi, Fukuoka 8300011 Japan; 2 Department of Diagnostic Pathology, Kurume University Hospital, 67, Asahi-machi, Kurume-shi, Fukuoka 8300011 Japan

**Keywords:** Presacral epidermoid cyst, Laparoscopic surgery, Developmental cyst

## Abstract

**Background:**

Complete resection of presacral epidermoid cysts is recommended due to the potential for infection or malignancy. Transsacral and transabdominal approaches have been used to treat presacral tumors. However, there are no standard surgical approaches to resection. We present the case of a presacral epidermoid cyst in an obese male patient who underwent laparoscopic transabdominal resection.

**Case presentation:**

A 44-year-old man was referred to our hospital for treatment of a cystic tumor on the pelvic floor. Contrast-enhanced computed tomography revealed a 45 × 40-mm tumor on the left ventral side of the rectum, right side of the ischial spine, dorsal side of the seminal vesicles, and in front of the 5th sacrum. Enhanced magnetic resonance imaging revealed a multilocular cystic tumor with high and low signal intensities on T2-weighted images. The tumor was diagnosed as an epidermoid cyst. We considered the transsacral or laparoscopic approach and decided to perform a laparoscopic-assisted transabdominal resection since the tumor was in front of away from the sacrum, and a transsacral approach would result in a larger scar due to poor visibility from the thickness of the buttocks. The entire tumor was safely resected under laparoscopic guidance, because the laparoscopic transabdominal approach can provide a good and magnified field of view even in a narrow pelvic cavity with small skin incisions, allowing safe resection of the pelvic organs, vessels, and nerves while observing the tumor contour.

**Conclusions:**

The laparoscopic transabdominal approach is an effective method for treating presacral tumors in obese patients.

## Background

An epidermoid cyst is a benign congenital tumor rarely found in the presacral cavity. Complete resection is recommended due to the risks of infection, fistula, and malignant transformation [1]. The deep pelvic cavity is narrow and full of blood vessels and vital nerves around the rectum; therefore, precise anatomical knowledge is required for surgery. Transsacral and transabdominal approaches have been used to treat presacral tumors. Recently, case reports on laparoscopic approaches have increased due to the advantages of magnified vision and accessibility to the pelvic floor [2, 3]. We selected the surgical approach considering the tumor location, size, and patient physique; however, it is still controversial which approach is better, and there is no standard for the selection of the surgical approach. Here, we present a case of a presacral epidermoid cyst in an obese man that was safely resected using a pure laparoscopic approach with good visibility.

## Case presentation

**Fig. 1:**
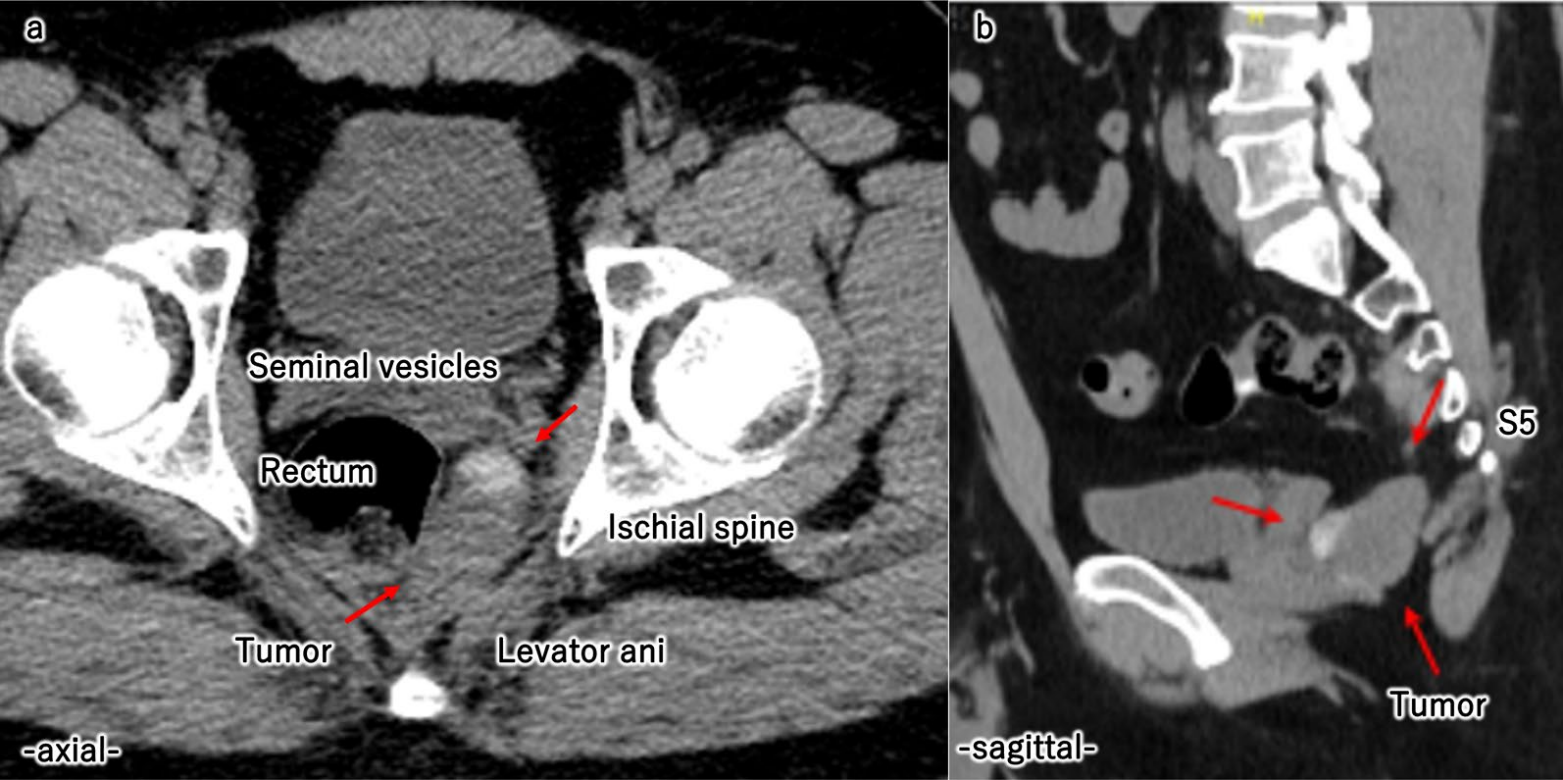
Abdominal contrast-enhanced CT. Contrast-enhanced CT showed a 45 × 40 mm tumor on the left ventral side of the rectum, the right side of the ischial spine, the dorsal side of the seminal vesicles (**a**), and anterior to the 5th sacrum (**b**)

**Fig. 2:**
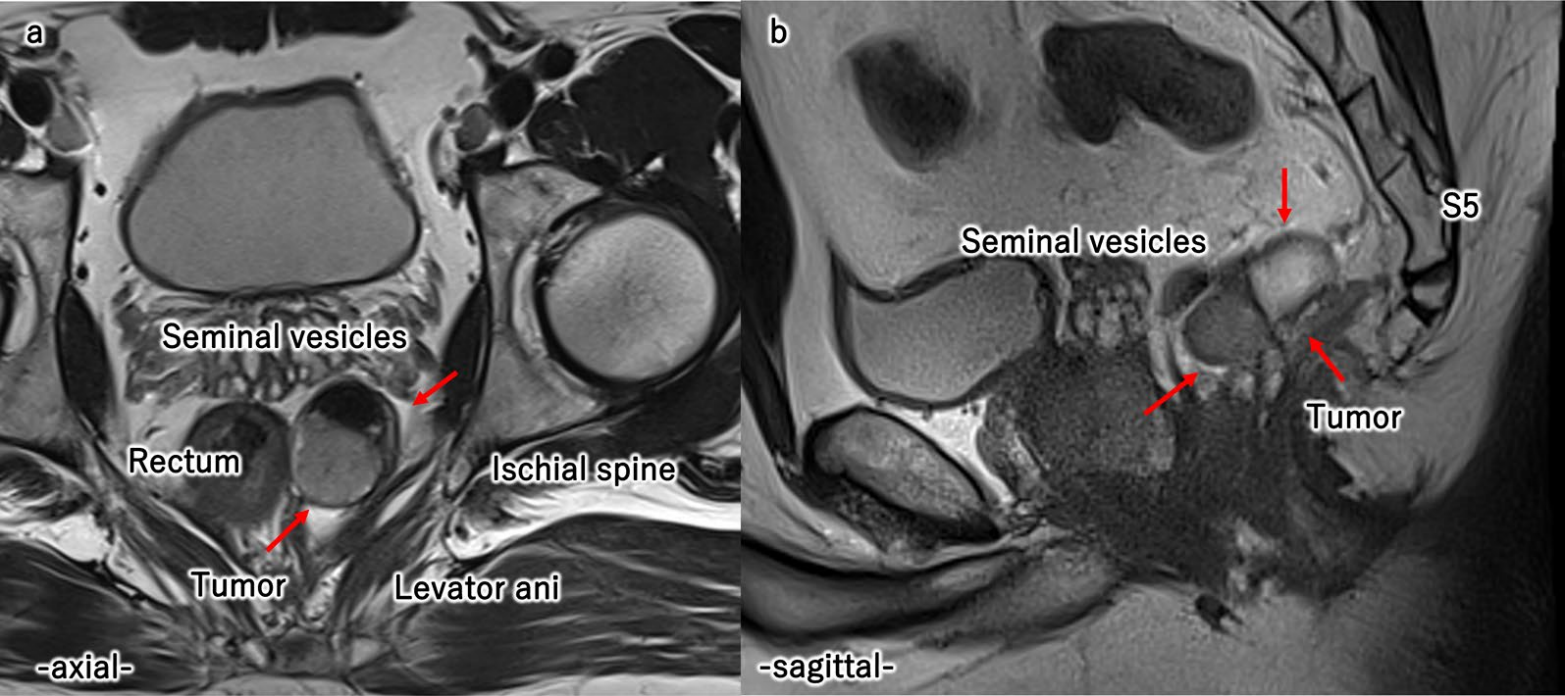
Abdominal MRI. The tumor contained highly and iso-dense areas. Enhanced magnetic resonance imaging (MRI) showed a multilocular cystic tumor with high and low signal intensity on T2-weighted images (**a**, **b**). The tumor had no contrast effect

A 44-year-old man was referred to our hospital for treatment of a cystic tumor on the pelvic floor. He had been followed up for fatty liver disease at another hospital, and a presacral cystic tumor was unexpectedly detected on computed tomography (CT). He was highly obese, with a body mass index of 36.4 kg/m^2^. The tumor was neither palpable on digital rectal examination nor detected on colonoscopy. Tumor marker levels were within the normal range. Contrast-enhanced CT revealed a 45 × 40-mm tumor on the left ventral side of the rectum, right side of the ischial spine, dorsal side of the seminal vesicles, and in front of the 5th sacrum (Fig. 1a, b). The tumor contained highly and iso-dense areas with no enhancement effects. Enhanced magnetic resonance imaging (MRI) revealed a multilocular cystic tumor with high and low signal intensities on T2-weighted images (Fig. 2a, b). The tumor was diagnosed as an epidermoid cyst based on the imaging findings, and the patient was scheduled to undergo surgical treatment. We considered the transsacral or laparoscopic approach and decided to perform a laparoscopic-assisted transabdominal resection since the tumor was in front of away from the sacrum, and a transsacral approach would result in a larger scar due to poor visibility from the thickness of the buttocks.

**Fig. 3:**
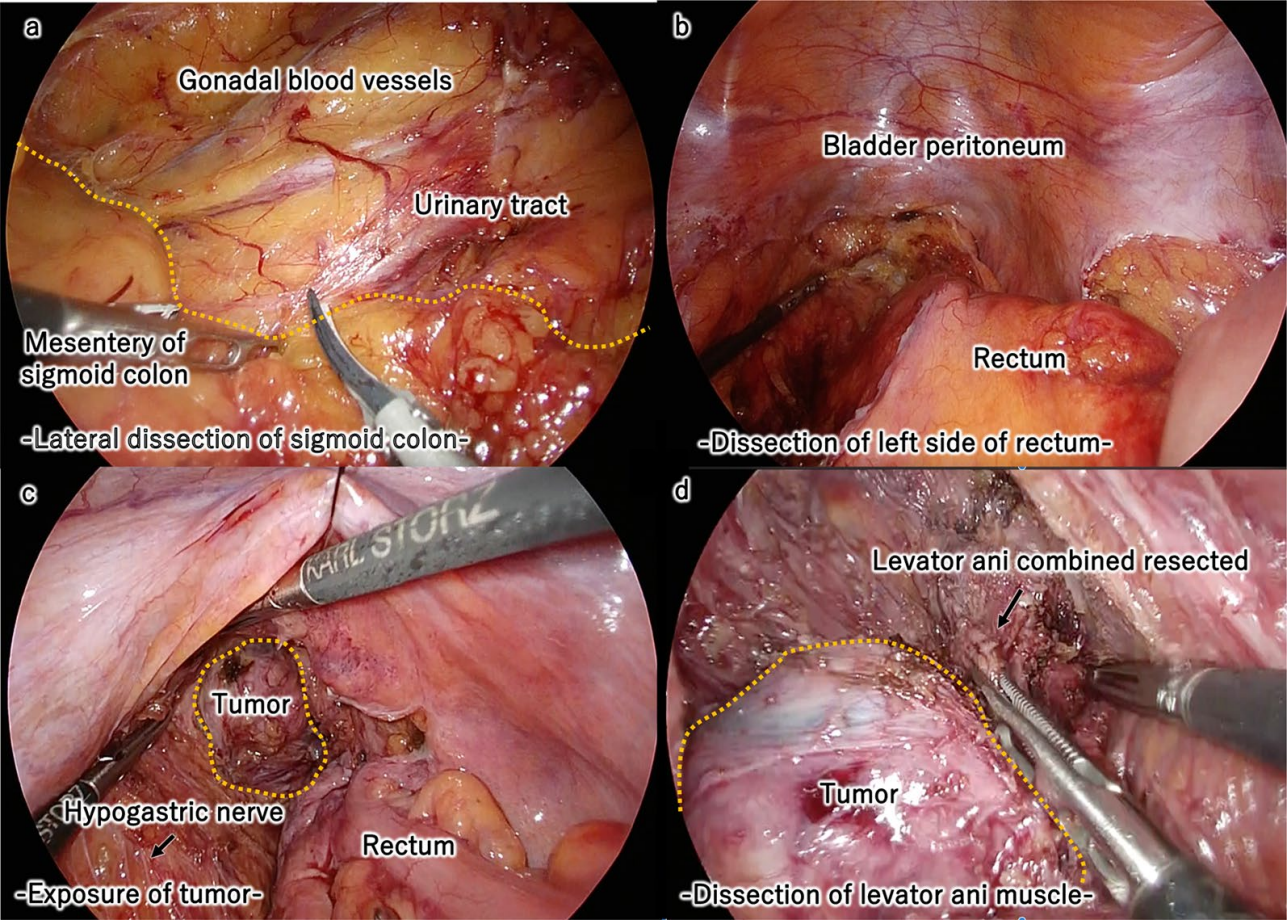
Intraoperative findings. We incised the peritoneum outside the sigmoid colon and entered into the Toldt’s fusion fascia (**a**). We dissected the left side of the rectum and proceeded to dissect the left side of the rectum preserving the hypogastric nerves, then reached a capsulated tumor in the presacral cavity (**b**). The sparse connective tissue around the tumor was dissected. The base of the tumor was attached to the levator ani (**c**). The levator ani was partially resected with the tumor attached, without injuring the rectum (**d**)

The patient was placed in the lithotomy position. A 12-mm camera port was placed at the umbilicus, followed by the placement of a 12-mm port in the lower right quadrant of the abdominal region, and three 5-mm ports in the bilateral upper and left lower quadrants. We incised the peritoneum outside the sigmoid colon and entered the Toldt’s fusion fascia (Fig. 3a). We dissected the left side of the rectum and continued the dissection of the left side of the rectum, preserving the hypogastric nerves, and reached a capsulated tumor in the presacral cavity (Fig. 3b). The sparse connective tissue around the tumor was dissected with the ultrasonic scalpel and the electric scalpel (Fig. 3c). The base of the tumor was attached to the levator ani. The levator ani was partially resected with the tumor attached, without injuring the rectum (Fig. 3d). We placed the tumor in a tissue storage bag, lengthened patient’s umbilical wound slightly, then collected the tumor. The tumor was safely resected under laparoscopic observation. The operation time was 286 min, and total blood loss was 18 ml.

**Fig. 4:**
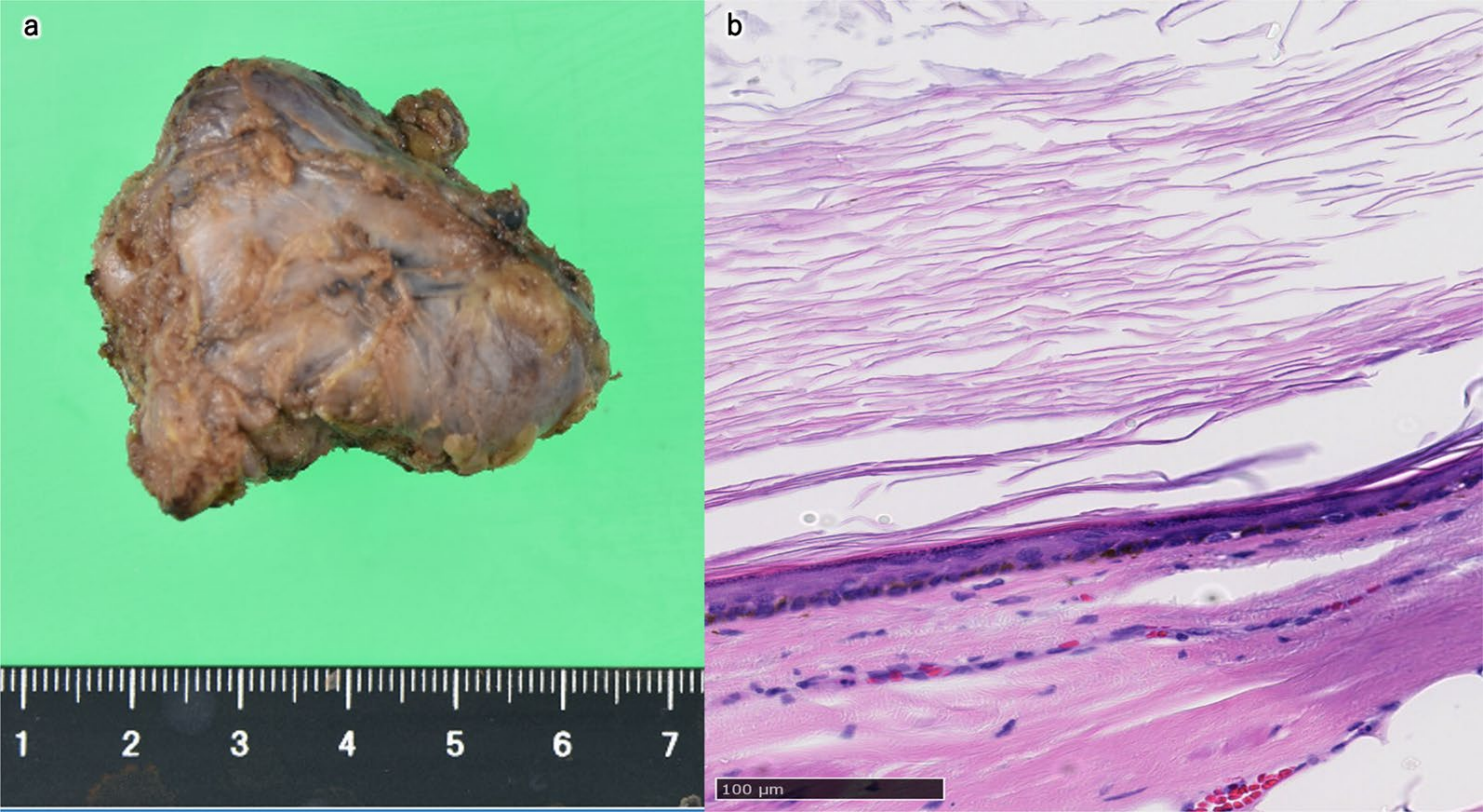
Macroscopic and histological examination. Macroscopically, the tumor size was 45 × 40 mm, and a cystic lesion containing yellowish-white atheromatous material was observed (**a**). Microscopically, the wall of the cystic lesion was composed of squamous epithelium with a granular layer and stratified keratin in the lumen (**b**)

Macroscopically, the tumor size was 45 × 40 mm, and the cystic lesion contained yellowish-white atheromatous material (Fig. 4a). Microscopically, the wall of the cystic lesion was composed of a squamous epithelium with a granular layer and stratified keratin in the lumen (Fig. 4b). The pathological diagnosis was epidermoid cyst. The tumor did not invade the anal levator ani; however, the muscles were still resected with the tumor. There were no malignant tumor components. Although he had slight gluteal discomfort after surgery due to partial resection of the levator ani, it resolved within two months, and there was no anal dysfunction. The patient was discharged on postoperative day 8 with good progress. There was no recurrence within one year, and there were no noted symptoms.

## Discussion

Presacral tumors are estimated to occur in 1 in 40,000 hospitalizations, and the majority of tumors are reported in female (77%–94%) [4, 5]. The presacral cavity is surrounded anteriorly by the rectum, posteriorly by the sacrum/coccyx, cranially by the peritoneal reflection, and caudally by the anterior sacrococcygeal ligament and coccygeus muscle [4]. Various tumors arise in the presacral cavity due to the contact with the caudal end of the fetus and the aggregation of numerous fetal tissues, and these have been defined as developmental cysts [1, 6]. Developmental cysts are classified as dermoid, epidermoid, and tailgut cyst [1]. Dermoid and epidermoid cysts are composed of a squamous epithelium with a cheese-like keratin component without fluid [1]. Dermoid cysts contain skin appendages that distinguish them from epidermoid cysts [1]. Tailgut cysts are defined as mucinous-secreting cysts due to the remnants of the tailgut [1]. Most cases are asymptomatic and are incidentally identified by CT or MRI [4]. Surgical resection is recommended since the tumor is associated with infection and has malignant potential, such as squamous cell carcinoma arising from a presacral epidermoid cyst [3, 4, 7].

Surgical approaches include transsacral incision, transabdominal (laparoscopic), or a combination of both. Transsacral incision is an easy way to access the tumor without laparotomy. However, there are risks associated with the need for a large wound over 10 cm in size, postoperative anal dysfunction, fistula formation, and sacral nerve disorder [8]. Previous reports have cited that transsacral incision is a useful method when the tumor is located below S3, near the coccyx, or in the ischiorectal space [6, 9]. The laparoscopic transabdominal approach can help recognize the rectum, surrounding blood vessels, and nerves, especially due to a laparoscopic magnifying view with small incisions, whereas other reports suggest that the laparoscopic transabdominal approach is preferable when the tumor is located above S3 and is larger than 8 cm [4, 9]. Disadvantages of the transabdominal approach include the risk of injury to the vessels or nerves around the rectum and abdominal adhesions after surgery. In consideration of our case, we selected a laparoscopic transabdominal approach although the tumor location was below S3 and the tumor size was less than 8 cm that the transsacral incision was acceptable, because the tumor existed anterior to the rectum, separated from the sacrum in contact with the levator ani, and the patient was severely obese. If the transsacral approach is selected, it may be difficult to reach the tumor when it is anteriorly separated from the sacrum. Additionally, the transsacral approach may require a large incision, poor vision, and a narrow working space in obese patients with large buttocks. We considered that transsacral approach is suitable for the tumor which location was below S3 and close to the anterior of sacrum in non-obese patients. We would like to emphasize that the choice of surgical approach should consider not only the location, extent, and size of the tumor, but also the patient’s physique. The laparoscopic transabdominal approach can provide a good and magnified field of view even in a narrow pelvic cavity with small skin incisions, allowing safe resection of the pelvic organs, vessels, and nerves while observing the tumor contour. However, surgery for obese patients takes more operative time, blood loss, and increases the postoperative complications compared with non-obese patients [10]. Laparoscopic surgery of obese patients is hard to move small intestine for securing a field of view, and identify the exfoliation layer because of rich visceral fat. It is important to apply proper traction and counter-traction to exfoliation layer to avoid accidental injuries of nerves and vessels. We should consider strengthening the head position and adding another port when the sufficient field of view or proper traction were not secured. We could also consider preoperative weight loss before surgery because the tumor had no findings of malignancy or infection. Recently, there has been growing evidence that minimally invasive surgery, such as laparoscopic or robotic surgery, can be used successfully for lesions below the S3 level, even in obese patients [11]. The patient experienced no postoperative anal dysfunction in our case.

## Conclusions

The laparoscopic transabdominal approach is effective for presacral tumors in obese patients.

## Data Availability

The datasets used in this report are available from the corresponding author on reasonable request.

## Abbreviations

CT: Computed tomography
MRI: Magnetic resonance imaging
